# Perspectives on strategies for improving ultra-deep desulfurization of liquid fuels through hydrotreatment: Catalyst improvement and feedstock pre-treatment

**DOI:** 10.3389/fchem.2022.807225

**Published:** 2022-07-22

**Authors:** Tendai O. Dembaremba, Siphumelele Majodina, Ryan S. Walmsley, Adeniyi S. Ogunlaja, Zenixole R. Tshentu

**Affiliations:** ^1^ Department of Chemistry, Nelson Mandela University, Gqeberha (Port Elizabeth), South Africa, Nelson Mandela University, Gqeberha, South Africa; ^2^ Research and Development Division, Sasol Technology (Pty) Ltd, Sasolburg, South Africa

**Keywords:** desulfurization, denitrogenation, crude oil, fuel refinery, hydroprocessing, hydroprocessing catalysts, adsorptive denitrogenation, metal-organic frameworks

## Abstract

Reliance on crude oil remains high while the transition to green and renewable sources of fuel is still slow. Developing and strengthening strategies for reducing sulfur emissions from crude oil is therefore imperative and makes it possible to sustainably meet stringent regulatory sulfur level legislations in end-user liquid fuels (mostly less than 10 ppm). The burden of achieving these ultra-low sulfur levels has been passed to fuel refiners who are battling to achieve ultra-deep desulfurization through conventional hydroprocessing technologies. Removal of refractory sulfur-containing compounds has been cited as the main challenge due to several limitations with the current hydroprocessing catalysts. The inhibitory effects of nitrogen-containing compounds (especially the basic ones) is one of the major concerns. Several advances have been made to develop better strategies for achieving ultra-deep desulfurization and these include: improving hydroprocessing infrastructure, improving hydroprocessing catalysts, having additional steps for removing refractory sulfur-containing compounds and improving the quality of feedstocks. Herein, we provide perspectives that emphasize the importance of further developing hydroprocessing catalysts and pre-treating feedstocks to remove nitrogen-containing compounds prior to hydroprocessing as promising strategies for sustainably achieving ultra-deep hydroprocessing.

## Introduction

Crude oil, and fossil fuels in general, were formed when large amounts of dead organisms buried under sedimentary rock were subjected to intense heat and pressure (Höök et al., 2010). It is predominantly composed of hydrocarbons that are desirable for energy needs (Sami and Hatch, 2001). Demand for petroleum is especially high in motorized transport, particularly long haul air and sea transport, due to its high volumetric density (Saleh, 2015). Consumption of crude oil rose from around 70 million barrels per day (Mb/d) in 1995 to 80 and 95 Mb/d in 2005 and 2015, and to over 100 Mb/d in 2019 (Statistical Review of World Energy, 2022). The overall share of renewable energy sources remains relatively low at 5.7% (Statistical Review of World Energy, 2022). To bridge the gap in adoption of renewables, legislation is being implemented to minimize fossil fuel based emissions, reduce their carbon footprint, and to develop strategies for CO_2_ capture, storage and utilization.

The hydrocarbon composition of crude oil is accompanied by several other elements such as oxygen, sulfur, nitrogen, and metals which give rise to most of the challenges associated with its handling, storage and use (Théodet, 2010). Sulfur and nitrogen pose the biggest challenges (Hansmeier et al., 2011). As a result, sweet crude oils (crude oils with lower contamintants, especially sulfur and nitrogen), are generally preferred compared to sour crude oils (crude oils with higher concentrations of contaminants). At the projected demands, the preferred sweet crude sources will soon be depleted forcing the switch to more sour crude sources (Campbell and Laherrère, 1998; Dincer and Zamfirescu, 2018; Bardi, 2019). More than 70% of the world’s oil reserves are classified to be of heavier and sourer composition (World Energy Review, 2020).

Due to the rising demand for “clean” energy, pressure is mounting for the world to adopt cleaner energy technologies and improve the use of fossil fuels. Crude oil refining will be with us for a while to come so it is crucial that we continue to develop the technology. Conventionally, hydroprocessing is used to remove sulfur-containing compounds. Several other methods such as oxidative desulfurization, biodesulfurization, extractive desulfurization, and adsorptive desulfurization are also being pursued. It has been difficult to replace hydroprocessing as the industrially preferred method. As such, this work provides perspectives on the advances that are being made in improving the hydroprocessing technology to achieve ultra-deep desulfurization. Emphasis is placed on hydroprocessing catalyst development and improvement of feedstock through pre-treatment to remove nitrogen-containing compounds.

### Sulfur- and nitrogen-containing compounds in crude oil

Sulfur and nitrogen content of crude oil is known to vary significantly depending on the source and there is no relationship between the concentrations of the two (Prado et al., 2017). The sulfur- and nitrogen-containing compounds are predominantly in aromatic form and generally increase with the heaviness of the crude source (Wiwel et al., 2000; Zeuthen et al., 2001). In addition to other factors, the sulfur and nitrogen content of crude oils determine their acceptability as feedstock in the refinery industry (Choudhary et al., 2008).

After carbon and hydrogen, sulfur is the next most abundant element in most crude oils (Javadli and de Klerk, 2012). Sulfur levels in crude oils are usually in the range 0.1–5.0 wt% and most have more than 1.0 wt% sulfur and are classified as sour; 0.5–1.0 wt%S is considered medium sour while <0.5 wt%S is considered sweet (World Energy Review, 2020). Sulfur-containing compounds are present in two forms; inorganic (*e.g*. suspended or dissolved elemental sulfur, hydrogen sulfide and pyrite) and organic (thiols and thiophenic compounds), the latter being the more abundant form (Javadli and de Klerk, 2012). Benzothiophenes and dibenzothiophenes are the most dominant sulfur-containing compounds in heavier fuels. Sulfur can also be found in combination with other heteroatoms and some sulfur-containing compounds may have more than one sulfur atoms. Sulfones and sulfoxides can also be found due to naturally occurring oxidation (Javadli and de Klerk, 2012).

Nitrogen levels in crude oil are generally lower compared to sulfur levels. About 90% of crude oils are classified as nitrogen poor, having less than 0.25 wt% nitrogen content while most are below 0.1 wt% (Prado et al., 2017). Four chemical classes of nitrogen-containing compounds are found in fuel oils: aliphatic amines, anilines, and two heterocyclic aromatic compound groups (five-membered pyrrolic and six-membered pyridinic compounds). Aliphatic amines and anilines are not present in significant amounts in most fuel oils (Wiwel et al., 2000). Aliphatic amines, anilines and six-membered pyridinic compounds are classified as basic nitrogen-containing compounds (pK_a_ ≥ 2) while the five-membered compounds are classified as neutral, non-basic or acidic (pK_a_ < 2) (Richter et al., 1952; Wiwel et al., 2000; Prado et al., 2017). The ratio of basic nitrogen to non-basic nitrogen remains within the range 0.25–0.35 irrespective of the origin of the crude oil (Prado et al., 2017). The most common basic nitrogen-containing compounds in fuel oils are pyridine, acridine, quinoline and their derivatives, and the most common neutral nitrogen-containing compounds are pyrrole, indole, carbazole and their derivatives (Asumana et al., 2011). In most cases, 75% of the total nitrogen content in the straight run gas oil and light cycle oil used as feed stocks for diesel fuel production consist of indole and carbazole type compounds while the 25% is constituted mainly of quinoline derivatives (Laredo et al., 2002; Prado et al., 2017). Heavier crudes (*e.g*. bitumen and tar sands) are associated with very high nitrogen content, mostly basic nitrogen-containing compounds, particularly quinoline derivatives (Wiwel et al., 2000; Javadli and de Klerk, 2012).

### Challenges associated with sulfur and nitrogen containing-compounds in liquid fuels

The primary concern of having sulfur-containing compounds in fuel oil is that upon combustion they produce sulfur oxides (especially sulfur dioxide) that have devastating effects on the environment, biodiversity and human health (Schlesinger and McQueen, 2010; Hansmeier et al., 2011; Thurston, 2017). Diesel engines operate at higher temperatures and pressures producing more sulfur oxide emissions than gasoline engines (Zolotareva et al., 2019). The gases aggravate the contribution of motorized transport to global warming as they poison catalysts required in vehicle emission control systems to minimize the escape of carbon monoxide (Hansmeier et al., 2011).

Sulfur- and nitrogen-containing compounds affect fuel storage and handling (Abro et al., 2016). Nitrogen-containing compounds cause storage problems due to instability and degradation which leads to formation of gums. Five-membered nitrogen-containing heterocycles such as pyrroles can easily undergo oxidative or thermal free-radical addition reactions to form heavy polymeric products commonly observed as gums or red tars (Abro et al., 2016). Sulfur-containing compounds are mostly responsible for promoting gum formation, stabilizing gum and may also react with some components of fuel oil to form materials of high molecular weight (Thompson et al., 1949). The gums formed are undesirable in storage tanks and tanks of vehicles, and negatively impact refinery processes and engine performance.

To make matters worse, sulfur and nitrogen-containing compounds interfere with the processes that are used to remove them from fuel oil. The compounds cause fouling of process equipment (Prado et al., 2017). They also inhibit the catalysts used during fuel refining processes such as hydrocracking and hydrotreatment, of which nitrogen-containing compounds are the main culprits (Sau et al., 2005). That makes sourer crudes difficult to process using conventional methods. High concentrations of refractory sulfur-containing compounds in sour crudes also persistently remain in the final product after most treatment procedures (Hansmeier et al., 2011; Forte et al., 2014).

It is evident that nitrogen-containing compounds are the ones that are mostly associated with the numerous challenges around the processing, handling, and storage of fuel. However, they are often “ignored” in favour of sulfur-containing compounds as they are usually much lower that sulfur-containing compounds and are removed simultaneously during hydrotreatment (Wiwel et al., 2000; Hansmeier et al., 2011; Prado et al., 2017). Considering the drive to achieve ultra-low sulfur levels of sulfur and also pave way for the utilization of sourer crudes, it is now imperative to find ways of removing even the small amounts of nitrogen-containing compounds from crude oil to prevent the inhibition of catalysts used in downstream processes such as hydrocracking and hydrotreatment.

### Regulation of sulfur and nitrogen levels in liquid fuels

Most governments have come up with strict legislations to curb the release of sulfur oxides into the environment, by primarily monitoring the amounts of sulfur in carburant fuels. The monitoring started as early as 1990 with specifications for different types of vehicles and designated areas (*e.g*. urban, highway, off-road) and the policies now apply across all vehicle types and areas (US: Fuels: Diesel and Gasoline, 2021; Fuel Regulation, 2022a; Fuel Regulation, 2022b). The most recent Clean Air Act (2021) from the United States Environmental Protection Agency requires liquid fuels to have less that 15 ppm sulfur content (Environmental Protection Agency, 2021). Euoropean Union sulfur limits are set under Euro VI at 10 ppm (ICCT, 2020). Brazil has standards equivalent to the Euro V but additionally banning the sale of diesel cars in the country while promoting ethanol and biofuel blending (Zolotareva et al., 2019; ICCT, 2022). From 2013, major metropolitan areas and selected stations around the country were able to supply 10 ppm diesel to trucks (ICCT, 2022). China’s vehicle emissions standards are also equivalent to the Euro V, being China V for light-duty vehicles and China V for heavy-duty vehicles (Zolotareva et al., 2019).

Ships have been lagging behind although some marine areas have been declared to be designated emission-controlled areas (along the shorelines) where ships are required to use light distillate fuels such as marine gas oil to reduce pollution levels. USA and Canada applied to the International Maritime Organization to establish emission control areas along their shorelines where the sulfur limit was 10,000 ppm from 2010 to 1000 ppm from 2015, marking the beginning of international regulation of sulfur limits in marine fuels (IMO, 2022). In 2020 the MARPOL convention introduced the so-called “IMO-2020” regulation which cut the sulfur content in the marine fuel from 3.5% m/m down to 0.5% m/m outside designated emission control areas. The sulfur specification is even lower at 0.1% m/m for designated emission-control areas.

**FIGURE 1:**
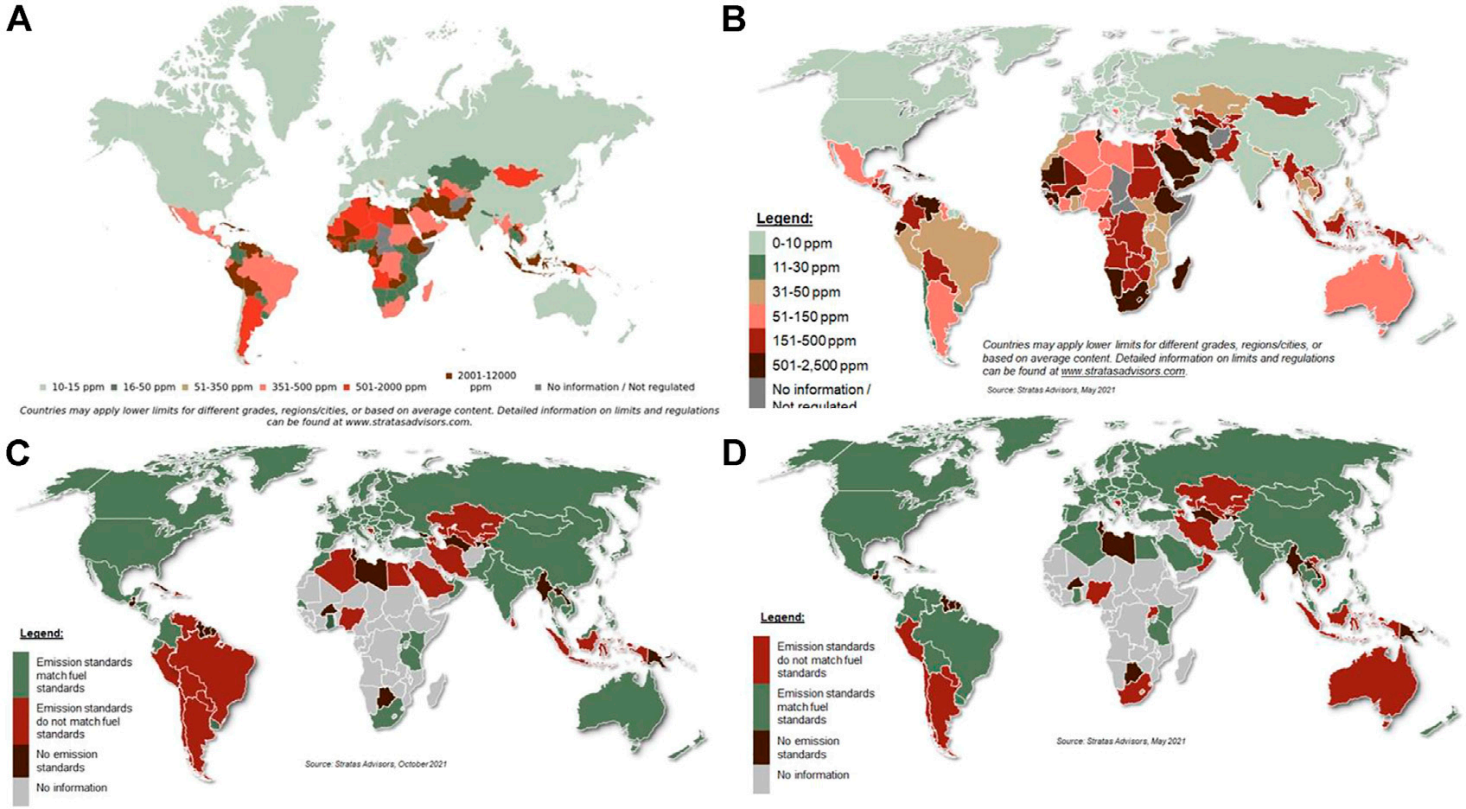
Maximum sulfur limits in **(A)** gasoline 2021 and **(B)** on-road diesel 2021, and gap between current vehicle emission standards and **(C)** gasoline quality and **(D)** on-road diesel quality (Global Fuel Specification, 2022).

African countries have also been lagging behind. Morocco was the pioneering country in Africa starting with 50 ppm diesel in 2009 and then 10 ppm diesel becoming available from 2011 (UNO, 2022). Other leading countries in adopting legislation for low sulfur levels in fuel Africa are Mauritius, Kenya, Uganda, Tanzania, Rwanda, Burundi, Ghana, Mozambique, Malawi and Zimbabwe (UNO, 2022). In Mauritius 50 ppm diesel had become a standard across all fuel stations from 2012. Mozambique implemented 50 ppm limit for gasoline in 2017. Zimbabwe implemented the 50 ppm limit for diesel in 2018 with a goal to progressively phase out 500 ppm diesel. However, despite African countries adopt the legislation for low sulfur diesel implementation remains a challenge as can be seen from Figure 1 (Global Fuel Specification, 2022).

Most of the discussed legislations such as the Clean Air Act of USA and the Euro 5/6 do not directly minitor the amounts of nitrogen-containing compounds in the curbarent fuel but rather control the emission levels of nitrogen oxides, particularly NO_2_. Nevertheless, it is important to have nitrogen levels below 10 ppm (Abro et al., 2016).

### Overview of crude oil processing

The crude oil refinery process is primarily meant to separate the hydrocarbons according to their boiling points (*viz*. molecular weight) through fractional distillation into fractions suitable for specific applications such as fuels, lubricants, and feedstock for other downstream petrochemical industries. Product improvement is also important during refinery and encompasses processes applied to adjust the boiling range of the products to target more valuable cuts or to introduce special properties that improve the product performance and to ensure adherence to given specifications (Speight, 2018). Common refinery boiling point adjustment technologies include alkylation (increase in boiling point) as well as fluid catalytic cracking and hydrocracking (boiling point reduction). Naphtha reforming and isomerization is used primarily to improve gasoline octane numbers, while hydrotreatment is typically used to remove heteroatoms and improve product stability (Speight, 2018). The main categories of fuel oils from the distillation of crude oil are heavy gas oil, diesel/light gas oil, kerosene and they serve as different carburants in different forms of motorized transport.

Removal of sulfur- and nitrogen-containing compounds is complicated by the complexity of the fuel oil matrix. (Hansmeier et al., 2011) Reductive or oxidative techniques are primarily used in industry to remove sulfur and nitrogen-containing compounds (Forte et al., 2014). The techniques can be applied before the use of the fuel oil or on exhaust fumes (Zolotareva et al., 2019). However, removal of sulfur- and nitrogen-containing compounds from exhaust fumes is more applicable to industrial plants. Practically, the techniques cannot be applied to motorized transport as a primary method for removing the noxious gases that come from the sulfur and nitrogen compounds. Catalytic converters can only sufficiently manage small quantities of sulfur oxides that come from low sulfur fuel oils. That still leaves the removal of sulfur- and nitrogen-containing compounds from oil fuel before use as the most preferred technique. In that regard, hydroprocessing has proven to be the more practical route due to how easily it can be applied on an industrial scale and has been the conventional technique (Huh et al., 2009). However, the technique has limitations and challenges that keep on motivating researchers to find alternative techniques or techniques to aid hydrotreatment.

### Conventional hydroprocessing

**FIGURE 2:**
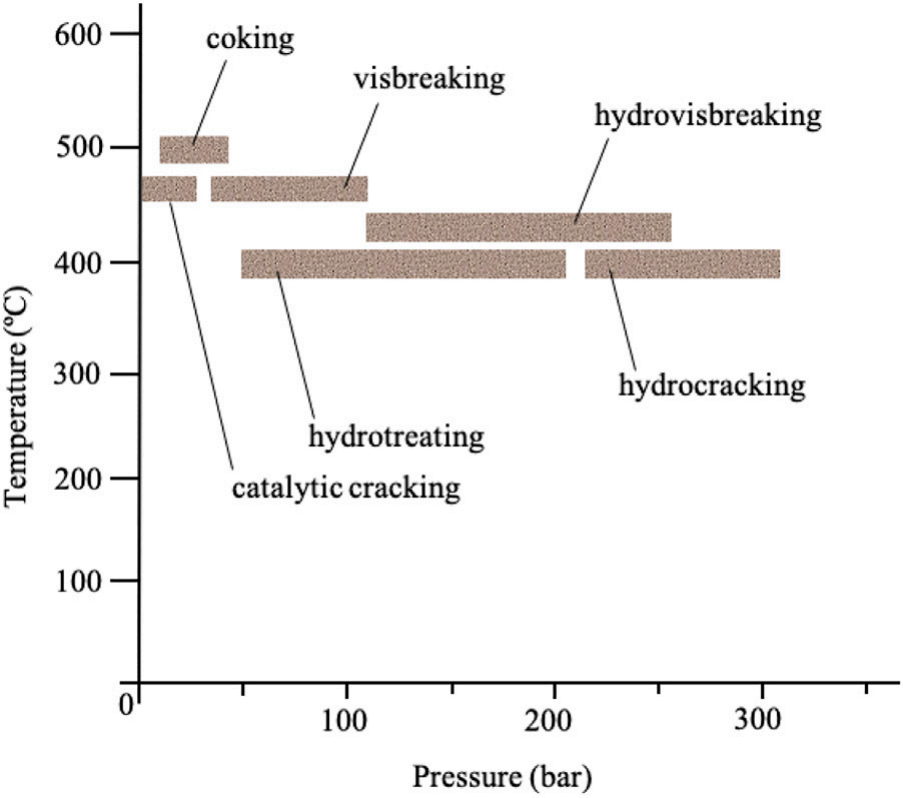
Hydroprocessing examples based on the process conditions (temperature and pressure) (Bose, 2015).

Hydroprocessing covers a range of catalytic processes such as hydrotreating, hydropyrrolysis and hydrocracking processes (Figure 2). This process is carried out prior to most processes (*e.g*. catalytic reforming) to alleviate the challenges associated with the heteroatoms found in fuel oil such as sulfur, oxygen, metals and nitrogen (Saleh, 2015). The removal of these unwanted heteroatoms is referred to as hydrodesulfurization (HDS) for removal of sulfur, hydrodenitrogenation (HDN) for removal of nitrogen, hydrodeoxygenation (HDO) for removal of oxygen, and hydrodemetalation (HDM) for removal of metals. The processes occur simultaneously (Hansmeier et al., 2011). During hydroprocessing, the sulfur and nitrogen in sulfur- and nitrogen-containing compounds react with hydrogen under high pressure (typically 15–90 bar) and high heat (typically 300–350°C) in the presence of catalysts to produce H_2_S and NH_3_, respectively, leaving behind the corresponding hydrocarbons (Huh et al., 2009; Hansmeier et al., 2011; Javadli and de Klerk, 2012; Zolotareva et al., 2019). Additional steps, such as the Claus process, are required to remove and convert H_2_S into elemental sulfur (Hansmeier et al., 2011; Saleh, 2015; Zolotareva et al., 2019).

**FIGURE 3:**
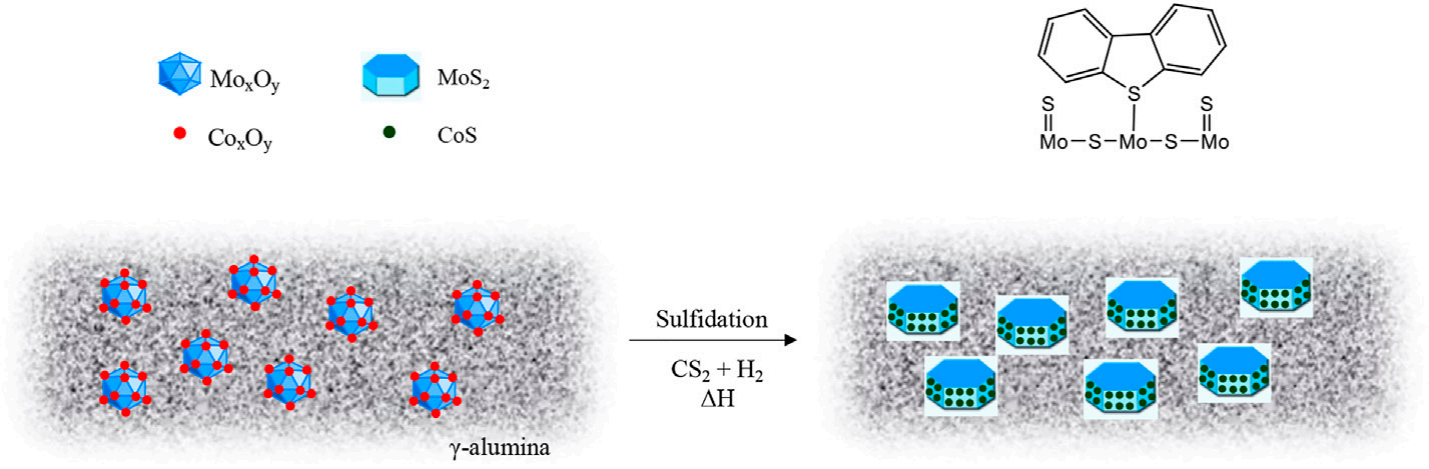
Schematic of the sulfidation process for CoMo catalyst showing DBT binding on a coordinatively unsaturated Mo site (Moqadam and Mahmoudi, 2013).

Hydrotreating catalysts consist of two or more metals, at least one being the active metal and the other one a promoter (Brunet et al., 2005). The most common hydrotreating catalysts contain the active metal as Molybdenum (Mo) or Tungsten (W), and Nickel (Ni) or Cobalt (Co) as the promoter metal. The most common hydrotreating catalysts are alumina supported Cobalt-Molybdenum (CoMo) and Nickel-Molybdenum (NiMo), being mainly used for HDS and HDN respectively. This is due to NiMo catalysts having higher hydrogenation activity than CoMo catalysts. The Nickel-Tungsten (NiW) catalyst has a higher denitrogenation activity than NiMo but is more expensive (Prado et al., 2017; Kaiser et al., 2020). The catalysts are commonly supplied in their air-stable oxide form and require sulfidation for activation (Figure 3). Sulfidation forms a metal-sulfide bond between the active metal sites in the catalysts and the sulfur compound used for sulfidation, usually carbon disulfide (CS_2_) or dimethyldisulfide. (Chowdari et al., 2020; Tanimu et al., 2021). The role of sulfidation is to form coordinatively unsaturated sites (CUS) or sulfur vacancies on the active metal where sulfur- and nitrogen-containing compounds are preferentially adsorbed by binding *via* the sulfur and nitrogen providing a driving force for their removal (Harris and Chianelli, 1986; Chianelli et al., 2020). In other words, the active forms of the hydroprocessing catalysts are actually transition metal sulfides.

### Conventional hydroprocessing challenges

Hydrotreatment does not completely remove sulfur- and nitrogen-containing compounds from fuel (Gray et al., 1995; Ma et al., 1996; Whitehurst et al., 1998). Reactivity is dependent on the molecular structure; paraffinic components, *i.e*. aliphatic acyclic sulfides (thioethers), cyclic sulfides (thiolanes), aliphatic amines and anilines can be easily removed while sulfurs and nitrogens in heterocycles are more resistant. The performance of hydroprocessing catalysts is negatively affected by steric and electronic factors of the heterocyclic compounds. Sulfur-containing compounds remaining in hydrotreated diesel fuels at sulfur levels lower than 500 ppm are predominantly dibenzothiophenes with alkyl substituents at the 4- and/or 6-position. Carbazole and its alkyl-substituted analogues are the most refractory nitrogen-containing compounds (van Looij et al., 1998; Wiwel et al., 2000; Zeuthen et al., 2001). The more the substituents in pyrrole benzologues the less their reactivity (Zeuthen et al., 2001). Carbazoles with methyl substituents at positions 1- and 8- were found to have extraordinarily low reactivity similar to 4,6-substituted dibenzothiophene. Interestingly, alkyl substitution at positions 4- and 5- also reduces reactivity significantly, inconsistent with the normally used steric restriction reasoning (Wiwel et al., 2000). Indoles and quinolines are very reactive compared to carbazoles (Zeuthen et al., 2001). For equivalent sulfur- and nitrogen-containing compounds (*e.g*. carbazole and dibenzothiophene), nitrogen-containing compounds have much lower reactivity (Zeuthen et al., 2001).

Small amounts of aromatic nitrogen-containing compounds in the fuel can easily inhibit the catalysts due to preferential adsorption (Zeuthen et al., 1991; Whitehurst et al., 2000; Koltai et al., 2002; Turaga et al., 2003; Wiwel et al., 2010; Hidalgo-Vivas et al., 2022). This has a serious negative effect on the kinetics of hydroprocessing, particularly removal of refractory sulfur-containing compounds. Basic nitrogen-containing compounds are considered the biggest problem in that regard as they severely inhibit hydrogenolysis and hydrogenation, steps that are crucial for hydroprocessing of refractory sulfur-containing compounds (Zeuthen et al., 1991; Whitehurst et al., 2000; Zeuthen et al., 2001; Koltai et al., 2002; Turaga et al., 2003; Wiwel et al., 2010; Prado et al., 2017; Hidalgo-Vivas et al., 2022). It has always been feared that even neutral nitrogen-containing compounds have a similar inhibitory effect since strongly basic intermediates (amines) are formed during hydrotreatment of neutral nitrogen-containing compounds (Forte et al., 2014; Prado et al., 2017; Wen et al., 2017). The final product of hydrodenitrogenation (NH_3_) is also strongly basic (Nakamura et al., 2005). An investigation by van Looij *et al.* (van Looij et al., 1998) shows that trace amounts of nitrogen compounds (0–30 ppm) have a significant inhibition effect to the reaction rate in deep hydrodesulfurization and are a key factor in achieving deep hydrodesulfurization of fuel oil while the influence of compositional changes *e.g*. content polycyclic aromatics is negligible. Sulfur-containing compounds and their hydrotreatment product H_2_S are also capable of the same, albeit to much a lesser extent (Nakamura et al., 2005; Kent, 2010). Neutralization depends on the equilibrium of the acid-base reaction which is determined by the strength of the acidic sites, the basicity of the compounds and temperature. Inhibitory effects are also higher at low temperatures (>350°C) (Prado et al., 2017). The more basic the compounds, the greater the inhibition.

Additionally, hydrodenitrogenation is an unfavourable process, it is a kinetically slow process, consumption of hydrogen is not stoichiometric (much higher quantities are required), and high amounts of ammonia affect the gas cleaning step and complicate other process aspects (Prado et al., 2017).

### Strategies for achieving ultra-deep hydroprocessing

Achieving ultra-low sulfur levels using conventional hydroprocessing is still a work in progress and a cause for concern for refiners when it comes to striking a balance between producing such products at reasonable market prices. There is now an increasing need for drastic improvements to the process to meet the required low sulfur specifications as well as accommodate the increasing heavier poorer feeds. Severe hydrotreatment conditions (higher temperature, residence time, pressure and more hydrogen) have been applied to try and remove the persistent sulfur- and nitrogen-containing compounds and also minimize inhibition of catalysts (Hansmeier et al., 2011; Prado et al., 2017). For example, hydrotreating of creosote distillate to produce a distillate blending material for diesel fuel is industrially performed at 280–380°C and 18.5 MPa (Prado et al., 2017). However, the use of severe operating conditions means: consumption of hydrogen is much higher, hydrodearomatization increases, and there is an adiabatic temperature increase since hydrodenitrogenation and hydrodearomatization are very exothermic reactions (Hansmeier et al., 2011; Prado et al., 2017). Often additives need to be added to manage the fuel specifications. Excessive use of hydrogen is also of concern due to increasing demand for hydrogen over the years, especially in green fuel technologies (Lima et al., 2019). Besides, the current production of hydrogen already has a huge carbon footprint (Javadli and de Klerk, 2012).

Better bets have been on catalyst development, better reactor designs and introducing new processing systems. (Lima et al., 2019). With regards to new processing systems, placement of different catalysts in multibed configurations so that they can work synergistically in what is known as catalyst stacking technology has been showing promise in improving hydroprocessing activity and reducing hydrogen consumption and other operating costs (Leal et al., 2022). The technology also allows expensive catalysts to be easily incorporated into the systems. However, the technology is still at its infancy, how it works is not yet fully understood and there are many factors to consider. Each optimal configuration is on a case-to-case basis as there is no established predictive method. However, just slight increamental gains are achieved at this stage.

Several complementary methods that can aid hydroprocessing to achieve ultra-low sulfur levels are widely reported in literature. These are mainly meant to target the refractory sulfur compounds and they oxidative desulfurization, biodesulfurization, extractive desulfurization and adsorptive desulfurization are the most popular. However, in this work our perspectives are only centered around further improvements that can be made on hydroprocessing catalysts and improving feedstock through pre-treatment to remove nitrogen-containing compounds.

## Improvements in conventional hydroprocessing catalysts

The development of novel hydroprocessing catalysts with excellent hydroprocessing performance is theoretically the most effective strategy as it can be easily adopted into existing fuel processing plants with minimal capital requirements (Zhang et al., 2019a). Hydroprocessing catalysts can be improved through new catalytic systems such as engineering new supports and/or new active phases (Huirache-Acuña et al., 2021). For example, replacement of transition metal sulfide active phases with noble metals has been successfully used. (Zhang et al., 2019a; Majodina et al., 2021). Ru and Pt as additives result in enhanced C-S cleavage activity as was demonstrated when they were added to a Rh-P catalyst and tested on 4,6-DMDBT (Kanda et al., 2022). Superior direct desulfurization selectivity has also been reported for Pt_2_Si/CNTs, Rh_x_Si/CNTs and RuSi/CNTs catalysts, with Pt_2_Si/CNTs having the highest activity, excellent stability and sulfur resistance (Yang et al., 2021). Small amounts of Ru were also shown to cause a significant increase in activity and selectivity for the products of the hydrogenation desulfurization pathways (Topalian et al., 2021). Highly active Re and Pd phases in Re/Pd-TiO_2_/SiO_2_ aerogel and xerogel catalysts have also been reported to result in higher catalytic activity compared to conventional CoMo catalysts (Prokić-Vidojević et al., 2021). However, the high cost and stability of platinum group metals discourages their industrial applications.

Even with the several hydrotreating catalyst improvements, refractory sulfur containing compounds continue to pose several challenges that make it difficult to achieve ultra-deep hydroprocessing. It is therefore important to take catalyst improvement strategies with an understanding that improved catalytic activity does not necessarily translate to completely treating refractory compounds in an economical way. Therefore, there is need for foundational changes to how the improvement of catalysts and the removal of sulfur- and nitrogen-containing compounds are looked at. Considering the several years of development of hydrotreatment catalysts, it has become more and more difficult to find major breakthroughs in catalyst improvement. Continous changes to the catalysts also means continuous changes to the industrial process conditions and these changes can be expensive, *e.g*. pressure increases/new reactors and increased saturation result in high exotherms and requirement for heat management or more hydrogen (cost). As such, the playing field still remains open for other potential strategies and research in those areas has been gaining traction in the past few decades. Considering that catalyst performance is also impeded by nitrogen-containing compounds, particularly basic nitrogen-containing compounds, there is also a need to develop hydrotreatment catalysts that take into cognizance those aspects. It is also important to explore the option of removing nitrogen containing compounds before hydrotreatment is carried out.

In this section, we explore the potential improvements that can be made on hydroprocessing catalysts to improve their catalytic activity and ability to hydrotreat refractory compounds. Inferrences can be made from groups of catalysts incorporating some of the proposed catalyst activity and selectivity strategies (Table 1).

**TABLE 1:** Typical improvements that are being made to improve the performance of hydroprocessing catalysts.

Catalyst	Chelating agent/Additives	Feed	Findings	References
PGM-containing catalysts				
RhMo/Al_2_O_3_, RhMo-x/Al_2_O_3_; x = EDTA, AA, CA)	EDTA, AA or CA	DBT	RhMo/Al_2_O_3_ (88%) achieved the higher HDS activity compared to chelated catalysts	Majodina et al. (2021)
Rh-M-P/SiO_2_; M (Ir, Pt, Ru)	P	4,6-DMDBT	The addition of Ru and Pt to Rh-P catalyst enhanced the C-S cleavage, with Rh-Pt-P catalysts having the highest HDS activity	Kanda et al. (2022)
Pt_2_Si/CNTs, Rh_x_Si/CNTs, RuSi/CNTs	—	DBT and 4,6-DMDBT	Superior selectivity to DDS pathway for deep HDS of DBT and 4,6-DMDBT was observed, with Pt_2_Si/CNTs having the highly HDS activity, excellent stability, and sulfur resistance	Yang et al. (2021)
Ni_2_P/SiO_2_, Ni_2-x_Ru_x_P/SiO_2_, Ru_2_P/SiO_2_	P	4,6-DMDBT	Ni_1.85_Ru_0.15_P/SiO_2_ catalyst was more active than Ni_2_P/SiO_2_ and Ru_2_P/SiO_2_ catalysts	Topalian et al. (2021)
Re/Pd-TiO_2_/SiO_2_	—	4,6-DMDBT	All catalysts achieved higher catalytic of 4,6-DMDBT than conventional CoMo catalysts	Prokić-Vidojević et al. (2021)
Co(Ni)Mo(W) catalysts with chelating ligands				
CoMo/γ-Al_2_O_3_	EDTA	2,6-dimethylanline, thiophene	EDTA led to an increase of promoted CoMoS sites with high HDN and HDS activity	Lélias et al. (2010)
NiW/Al_2_O_3_	CA	4, 6-DMDBT	More N-W-S phase formed and higher HDS activity was observed	Li et al. (2011)
NiMo/SiO_2_-Al_2_O_3_	EDTA	Straight run gas oil (SRGO)	EDTA reduced metal-support interaction and high HDS activity observed	Al-Dalama and Stanislaus, (2011)
CoMo/Al_2_O_3_	CA, TEG	DBT, SRGO	An increase in catalysts activity in HDS DBT and HT SRGO was observed	Budukva et al. (2019)
NiMo/ZrO_2_-TiO_2_	EDTA, CA	DBT	EDTA and CA catalysts showed superior to a NiMo/ZrO_2_–TiO_2_ formulation prepared with no organic additive	Escobar et al. (2008)
NiMo/SBA-15	EDTA, CA	DBT	Better dispersion was observed, highly active catalysts were obtained	Peña et al. (2014)
Co(Ni)Mo(W) with additives				
NiMo/γ-Al_2_O_3_	B and P	diesel fuel	Addition of B/P showed was more effective on HDS compared to HDN process	Soltanali et al. (2020)
CoMo/γ-Al_2_O_3_	P	Straight run gas oil	Inhibition behaviour of N compounds were observed	García-Gutiérrez et al. (2014)
CoMo/Al_2_O_3_	P	DBT	Lower metal support interaction, and increase in HDS activity was observed	Vatutina et al. (2019)
NiMo/γ-Al_2_O_3_	P	DBT and Quinoline	High dispersion of NiMoS, and high HYD selectivity observed, with 1.2 wt% P catalyst the best	Xiang et al. (2011)
NiMo/γ-Al_2_O_3_	B	DBT, 4,6-DMDBT	Maximum HDS of DBT and 4,6-DMDBT was obtained between 3 and 5 wt% B loading	Saih and Segawa, (2009)
NiMo/γ-δ-Al_2_O_3_	P and B	Straight run heavy VGO	Catalyst with B resulted in decrease in activity. The addition of P increased catalytic activity	Nadeina et al. (2019)
NiMo/HMS	P	4,6-DMDBT	P favours the sulfidation degree of Co species, creation of medium strength acid sites, and enhance 4,6-DMDBT HDS.	Nava et al. (2011)
CoMoW/γ-Al_2_O_3_	P	Coker light gas oil	High number of active sites, new Bronsted acid sites and enhanced HDN activity was obtained	Sigurdson et al. (2008)
Ternary catalysts				
CoMoW/Al-SBA-16	—	DBT	All catalysts showed high selectivity towards biphenyl, high Al-loading resulted in highest HDS activity	Huirache-Acuña et al. (2015)
NiMoW/clay hybrid	—	DBT and industrial kerosene	Uniformly dispersed NiMoWS particles were observed with high HDS activity and long-term catalytic stability	Liu et al. (2020a)
CoNiMo/γ-Al_2_O_3_	—	DBT	5% loading of Ni improved the HDS reaction. Increase in Ni decreased the DBT HDS activity	Cervantes et al. (2020)
NiMoW/SBA-16	—	DBT	The best HYD selectivity was archived by Ti *via* post-synthesis method	Alonso-Pérez et al. (2021)
NiMoW/Ti-HMS	—	DBT	The HDS reaction *via* the HYD pathway was increased by the presence of Ti	Huirache-Acuña et al. (2021)
Heteropolyacids (HPAs) as metal precursors				
Mo_n_W_12-n_S_2_/Al_2_O_3_	—	DBT, HYD naphthalene	Mixed MoWS_2_ phase and about 90% sulfidation of W and Mo was obtained	Kokliukhin et al. (2021a)
NiMoW/γ-Al_2_O_3_	—	DBT, HYD naphthalene and SRGO	Increased sulfidation of W and Mo, formation of highly active NiMoWS sites, and high activity was observed	Kokliukhin et al. (2021b)
Co-MoP/MCM-41-Al_2_O_3_	—	DBT, HYD naphthalene	mesoporous silica incorporation into Al_2_O_3_ improved morphological properties of the CoMoS active phase as well as the overall HDT activity	Glotov et al. (2021)

AA, acetic acid; CA, citric acid; TEG, triethylene glycol; EDTA, ethylenediaminetetraacetic acid; P, phosphorous; B, boron.

### Catalyst support considerations

The primary use of the support is for dispersing the active phase of the catalyst and to stabilize the catalysts. However, supports also influence the catalytic activity in various ways: by influencing the morphology of the active phase, modifying the electronic properties, and participating in bifunctional reactions with acid sites. A good support material has high surface area, optimum pore diameter (5–15 nm), optimum acidity, and moderate metal-support interaction (Shi et al., 2020). Different support materials have different effects on catalytic activity due to their distinct textural and structural properties, and the extent to which they interact with the active metal. Several studies have been conducted on a variety of supports such as TiO_2_, ZrO_2_, silica-alumina and mixed oxides (Duchet et al., 1991; Grzechowiak et al., 2001). It was discovered that TiO_2_ and ZrO_2_ show high activity (Caero et al., 2003). However, their low surface areas and small pore sizes make it difficult to disperse active phases and prevent diffusion of large bulky molecules onto the active sites leading to pore blockage and a decrease in catalytic activity (Michaud et al., 1998). The textural properties (morphology), affordability, and thermal and chemical stability, high tolerance for longevity, and good regeneration capacity of alumina supports (mainly ɣ-Al_2_O_3_) make them more advantageous and they are the most popular in hydrotreatment catalysts.

The chemical nature of the support material dictates the nature of the active phase. Strong metal-support interaction on the alumina support in Co(Ni)Mo-Al_2_O_3_ catalysts results in the formation of tetrahedral Mo oxides impeding full sulfidation. Due to this, research has focused on obtaining the optimum metal-support interaction, and this can be achieved by developing new supports for HDS and HDN application, or it can be engineered by introducing chelating ligands (Huirache-Acuna et al., 2013; Zhang et al., 2019b). The support needs not to be too acidic or too basic, since too much of either might lead to poor metal-support interactions resulting in poor Mo dispersion (Valencia and Klimova, 2011). However, it is important to note that high acidity leads to other undesirable side reactions such as coke poisoning and blockage of the active phase by basic nitrogen compounds (Alsolami, 2022). The support also has basic sites that contribute to the activity of the catalyst. Basic sites result in better dispersion of MoO_4_ and lead to low coke formation. Therefore, optimum metal-support interaction is desired to obtain proper sulfidation and dispersion of active species and to generate more Type II active phases. Type II active phases are the more active CoMoS phases formed from weak metal-support interactions. Weaker tetrahedral metal-support interactions allow for easier reduction and sulfidation process of molybdenum (tungsten) oxide unlike strong interactions that result in the formation of octahedral metal oxides which are difficult to reduce and sulfide resulting in the formation of the less active Type I phases.

**FIGURE 4:**
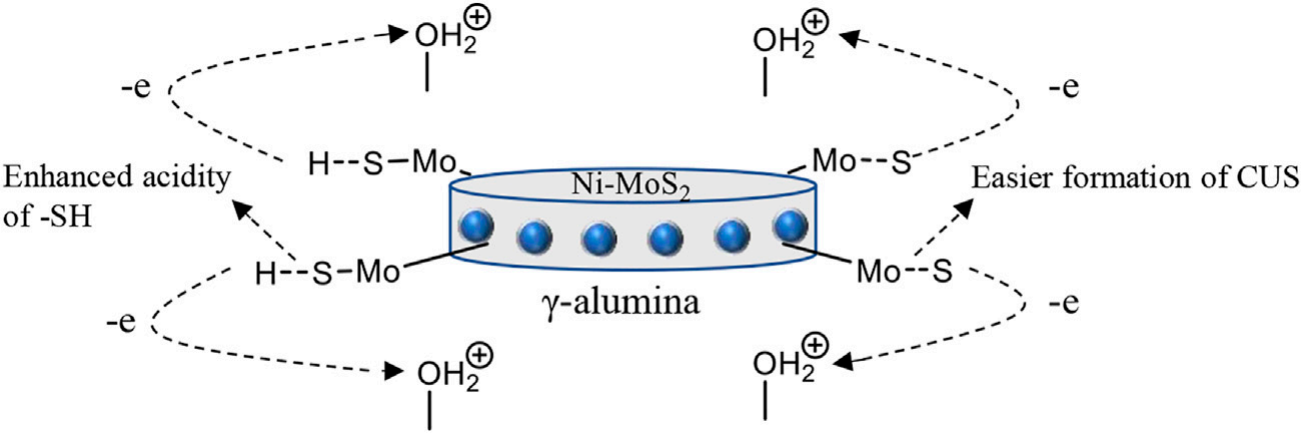
A representation of an alumina supported catalyst showing the movement of electrons on the active sites.

The morphology of the alumina support is crucial in determining the physicochemical properties that influence the catalytic behavior of the catalyst. The acidity of the alumina support is one of the most important features that controls the dispersion of Co(Ni)Mo and also affects the metal-support interaction (Chen et al., 2013). Brönsted acid sites found in the support material impart electronic effects on the active sites making it easier for coordinatively unsaturated sites to be formed when sulfur is released as H_2_S. They also act as a source of H_2_ to push towards the hydrogenation pathway through the S-H group found on the S-edge sites located on the active site of the catalyst (Figure 4). (Wu et al., 2014; Cao et al., 2020) Brönsted acid sites help to overcome the steric hindrance of alkyl DBTs through isomerization thus enhancing HDS activity (Breysse et al., 2003; Breysse et al., 2008). They also enhance C-N cleavage during denitrogenation since the denitrogenation process is linked to the S-H groups around the active site. Therefore, increased hydrogenation activity also results in an overall increased in HDS and HDN activity.

### Bimetallic vs*.* ternary catalyst considerations

Conventional bimetallic catalysts are susceptible to thermal, chemical, or mechanical degradation and metal coke poisoning, which often leads to inefficient ways to treat feedstock (Cervantes et al., 2020). This has motivated the development of trimetallic catalysts such as NiMoW, CoNiW, CoNiMo to increase the versatility of catalysts such as working in various harmful conditions and increased activity due to the additional active phase (Cervantes et al., 2020). These trimetallic catalysts have the advantage of having mixed phases [Ni(Co)MoWS] and their systems can be easily tailored compared to bimetallic and monometallic catalysts (Tanimu and Alhooshani, 2019). However, the catalyst performance results from different literature sources are contradictory. Severino *et al.* (Severino et al., 2000) and Sadeghbeigi (Sadeghbeigi, 2020) concluded that CoNiMo catalysts are less active as compared to bimetallic catalytic systems when they tested the HDS of thiophene and of 4,6-DMDBT, respectively. Mozhaev *et al.* (Mozhaev et al., 2016) and Cervantes *et al.* (Cervantes et al., 2020) obtained higher HDS activity for DBT when using CoNiMo catalysts compared to bimetallic catalysts. Higher catalytic activity for the trimetallic catalysts was also obtained for the desulfurization of heavy gasoline (Badoga, 2015). Monodispersed γ-Al_2_O_3_ supported NiMoW nanocatalysts also showed higher activity compared to NiMo and NiW bimetallic nanocatalysts when tested by Singh *et al.* (Tanimu and Alhooshani, 2019) With such contradictory results, it is important to note that the rational interpretation of the positive or negative role devoted to the addition of a second promoter has not been proposed yet (Cervantes et al., 2020). Therefore, more research can be focused on this work to improve the performance of the catalyst (Singh et al., 2021).

Unsupported versions of trimettalic catalysts have also been explored in what is known as NEBULA technology (Mendoza-Nieto et al., 2013). NEBULA catalysts predominantly contain metal sulfides in forms that incorporate porosity and avoid the use of catalyst supports to circumvent limitations in catalyst activity that comes from the limit in the amount of active metal that can be deposited on the pore walls of the support material. The catalysts show more superior HDS and HDN activities (about three times higher) than conventional alumina supported catalysts (Stanislaus et al., 2010). This is attributed to the metallic character, *i.e*. the formation of new mixed NiMoWS active phase which has better properties compared to conventional bimetallic NiWS, NiMoS, and CoMoS active phases. They also have better porosity character which is a limiting issue in supported catalyst. They are not limited by base interactions from the support and they contain a greater proportion of the formation of more Type II active phases. They contain more active metal per given volume with greater concentration of highly active Type II phases. The main disadvantage with Nebula catalysts is their high cost due to high metal content (Mendoza-Nieto et al., 2013). A study by Liu *et al.* (Liu et al., 2020b) using unsupported NiMoW catalyst on DBT showed a significant improvement of HDS activity but it was difficult to maintain the overall layered structure of the catalyst at high temperatures.

The success of NEBULA catalysts inspired the Albemarle-ExxonMobil research team to discover a new range of catalysts which they named Celestia catalysts (Leliveld and Slettenhaar, 2022). Celestia catalysts have higher catalytic activity than NEBULA catalysts. They have improved HDS performance and increased diesel yield enabled by turning down the cracking activity while still meeting product sulfur targets. The distillate and gasoline product have improved qualities and overall volume swell due to its exceptional saturation ability because it provides a higher exotherm. Celestia catalysts also provide the option to process more challenging feeds in hydrocracking pre-treatment without sacrificing unit length through increased catalyst deactivation. However, celestia catalysts require longer hold time to sulfide the catalyst fully. Their efficieny is best realized in high pressure applications. They are also typically very expensive. They are heavier (denser) than normal catalysts which is sometimes prohibitive when the equipment design limits are exceeded.

### Fine-tuning the active phases of the catalysts

#### The use of chelating ligands

The catalytic activity mostly depends on the formation of the active phase and its structure. Research has shown that during the activation of the catalyst the promoter sulfides first, *e.g*., Co or Ni (50–150°C) and then the Mo sulfides later from 175°C (Kishan et al., 2000; Coulier et al., 2001). The formation of the active phase requires the presence of a promoter (Co or Ni). Considering that the promoter sulfides first to form inactive phases (Co_x_S_y_ or Ni_x_S_y_), it is important to create a way of preserving promoter ions for the formation of the active MoS_2_ phase which only happens after the sulfidation of Mo (Kibsgaard et al., 2010). When there are fewer promoter ions remaining after the sulfidation of Mo there will be less chances of the promoter ions locating the MoS_2_ resulting in fewer CoMoS active phases forming and ultimately the formation of a catalyst with poor activity. Addition of chelating ligands during catalyst synthesis decreases the interaction of the promoter and the support by forming a promoter ion-chelating ligand complex which later decomposes to react with MoS_2_ to form the CoMoS active phases (Sundaramurthy et al., 2005). Most promoter ion-chelating ligand complexes only start to decompose above 200°C preventing early sulfidation of the promoter ions. Chelating ligands that have been applied for improving the formation of active phases include citric acid (CA), ethylenediaminetetraacetic acid (EDTA), glycol, nitriloacetic acid (NTA) (Sun et al., 2003). The addition of ligands also leads to the formation of polymolybdates in the oxide phase which are easily reducible, and metal dispersion is favorable during sulfidation of both cobalt and molybdenum oxides as they will start sulfiding at similar temperatures, thus resulting in the formation of more active phases (Badoga et al., 2012). The nature of the chelating ligand also imposes changes on the catalyst such as thermal stability, modulate the metal-support interaction, improve the dispersion of metal sulfides, and acidity leading to the formation of more CUS in the catalyst (de León et al., 2010; Rashidi et al., 2013; Zhang et al., 2016). Some of the advances that have been made in the use of chelating ligands to improve catalyst activity were summarized in Table 1.

A comparative study was carried out on HDS with catalysts synthesized on SBA-15 support with and without EDTA as a chelating ligand (Badoga, 2015). The sulfided catalysts were CAT 0 (NiMo/SBA-15), CAT 1 (NiMo/SBA-15, EDTA/Ni molar ratio 1), and CAT 2 (NiMo/SBA-15, EDTA/Ni molar ratio 2). A NiMo/Al_2_O_3_ catalyst (with no EDTA) was used for benchmarking. It was observed that all chelated catalysts had greater HDS catalytic activity compared to the standard NiMo/Al_2_O_3_ catalyst. The sulfur conversion followed the order: CAT2 (82%) > CAT1 (75%) > CAT 0 (70%) > NiMo/γ-Al_2_O_3_ (68%). Pena *et al.* (Peña et al., 2014) conducted a study on CoMo-SBA-15 catalysts with different amounts of MoO_3_ and CoO_3_ (Table 2). Benchmarking with CoMo6 and CoMo12, the chelated catalysts showed higher activity in the hydrotreatment of DBT in the order EDTA chelated catalysts > CA chelated catalysts > non-chelated catalysts.

**TABLE 2:** Catalysts with and without chelating agents and their overall DBT conversions.

Catalysts	Chelating agent	MoO_3_ (wt%)	CoO_3_ (wt%)		DBT conversion (%)	
					4 h	8 h
CoMo6	—	6	1.5	4:1	15.5	37.4
CoMo12	—	12	3	4:1	25.2	53.2
CoMo6E	EDTA	6	1.5	4:1	33.9	61.9
CoMo12E	EDTA	12	3	4:1	38.4	76.5
CoMo18E	EDTA	18	4.5	4:1	47.6	91.6
CoMo6C	CA	6	1.5	4:1	22.2	42.8
CoMo12C	CA	12	3	4:1	30.5	61.5
CoMo18C	CA	18	4.5	4:1	35.2	77.4

EDTA, ethylenediaminetetraacetic acid; CA, citric acid.

Majodina *et al.* (Majodina et al., 2021) recently tested a series of catalysts with and without chelating ligands tested for DBT HDS (RhMo_x_/Al_2_O_3_, x = EDTA, acetic acid, citric acid) and observed the following: RhMo/Al_2_O_3_ (88%) > RhMo-AA/Al_2_O_3_ (73%) > RhMo-CA/Al_2_O_3_ (72%) > RhMo-EDTA/Al_2_O_3_ (68%). The observed trend is contrary to the previous studies carried out on base metal catalysts where the importance of delaying sulfidation of the promoter (Co or Ni) sulfide, using chelating liagnds, was demonstrated. This opens up more questions around where chelation can be advantageous or how it can be applied for the different metals.

#### The use of additives

The addition of additives such as boron and phosphsorus also greatly improves the catalytic activity of conventional HDS and HDN catalysts (Table 1) (Rashidi et al., 2013; Vatutina et al., 2019; Soltanali et al., 2020). The addition of boron or phosphorous helps improve the acidity of the catalyst and the dispersion of the active phase. The acidity of the catalyst helps sterrically hindered refractory compounds to access the active sites. When Soltanani *et al.* (Soltanali et al., 2020) used phosphorus as an additive they additionally found that phosphorous can act as a promoter atom, helping with the distribution of MoS_2_ as it reduces Mo(VI) to Mo(IV). They also realized that phosphorous encourages the formation of octahedral Ni and Co structures vs*.* tetrahedral structures that cause polymerization of Mo-S bonds. Phosphorous also improves the degree of sulfidation of Co or Ni particles by reducing the metal support interaction. Additionally, phosphorous improves the formation of Brönsted acid sites on the catalyst, hydrogenation of aromatic rings, stability of the impregnation solution resulting in better metal distribution on the support, and the thermal stability of alumina. It also prevents the formation of inactive NiAl_2_O_4_ particles during catalyst synthesis and coke formation during hydrotreatment (Poulet et al., 1993; Jian and Prins, 1998; Maity et al., 2008; Rayo et al., 2012; Rashidi et al., 2013; Soltanali et al., 2020). The same properites can also be obtained using boron; increasing dispersion of the active phase, modifying the acidity of the support and reducing metal-support interaction.

#### Changing synthesis strategies (using different precursor compounds)

Replacement of conventional active metal precursors with precursors having two or more metals in the form of heteropolyacids (HPAs) in the preparation of oxidic precursors of metal-sulfide catalysts enhances catalyst activity (Ramírez et al., 2012; Minaev et al., 2015). Starting with such multimetal precursor compounds results in all metals being in close proximity allowing them to interact better with each other. Keggin-type HPAs such as B-H_4_[SiMo_3_W_9_O_40_] have been used as precursors for alumina supported catalysts giving the advantage to introduce simultaneously both metals in the same compound allowing to maintain a Mo-W nanoscape proximity (Nikulshina et al., 2018; Nikulshina et al., 2020) obtained Mo_x_W_1-x_S_2_ active phases using SiMo_n_W_12-n_ HPA precursors and observed higher catalytic activity for DBT (Nikulshina et al., 2018). Higher hydrodrogenation activity for naphthalene using the same catalyst was also observed by Nikulshina *et al.* (Nikulshina et al., 2020) Monometallic HPAs have also been investigated and it was found that the use of a mixture of H_4_SiMo_12_O_40_ and H_4_SiW_12_O_40_ leads to preferential formation of corresponding MoS_2_ and WS_2_ active phases, respectively (Kokliukhin et al., 2021a). Thomazeau *et al.* (Thomazeau et al., 2007) reported that the formation of mixed MoWS_2_ crystallites is possible only from a precursor which contains both closely related metals in the structure at once. The introduction of HPAs as precursors has also inspired a new method for preparing trimetallic catalysts (Kokliukhin et al., 2021a).

Metal borides, phosphides, carbides, and nitrides have also been investigated as metal precursors (Alexander and Hargreaves, 2010; Prins and Bussell, 2012; Carenco et al., 2013; Topalian et al., 2021). Transition metal phosphides (Ni_2_P, Co_2_P, MoP) have been found to produce highly active phases that lead to high HDS and HDN activity (Cecilia et al., 2009; Shamanaev et al., 2021). Ni_2_P gives the highest activity and performs better when it comes to hydroprocessing refractory compounds (Miles et al., 2020; Jiang et al., 2021). Metal phosphides optimize the acidity of the material support, positively affecting the dispersion of the active phase resulting in improved catalytic performance (Cecilia et al., 2009). Metal phosphides have high thermal and electrical conductivity, high thermal stability, and are resistant to the inhibition of sulfur- and nitrogen-containing compounds under standard hydrotreating conditions (Yun and Lee, 2014; Topalian et al., 2021). However, Jiang *et al.* (Jiang et al., 2021) investigated Ni_2_P catalysts and found contradictory results that showed challenges with dispersion, limited total HDS activity and instability. Transition metal carbides and nitrides such as molybdenum carbides and molybdenum nitrides have simple crystal structures that possess similar properties to those of transitions metals normally used for hydroprocessing catalysts, *e.g*. desirable electronic and magnetic properties, extreme hardness, and ionic charactericsts (Chen, 1996). However, they have additional advantages of being resistant to sintering at high temperatures, having strong interactions with nitrogen, sulfur and other heteroatoms in fuel and not causing C-C bond scission which leads to undesirable saturation of aromatics (Lin et al., 2021; Yue et al., 2021). They also have noble metal-like properties that make them effective hydrogenation catalysts (Yue et al., 2021). However, transition metal carbides and nitrides do not sustain long-term stability, they are easily deactivate due to their strong affinity with the adsorbates, especially at high temperatures (Lin et al., 2021).

The catalyst selection for ultra-deep desulfurization would be pre-mature at this stage as there is a lot of research work being undertaken. It is possible that a mix of catalysts, including those containing PMGs, may be a way to go in the application of the catalyst stacking technology.

## Removal of nitrogen-containing compounds prior to hydrotreatment

It is clear from the discussion in Section 1.6 that nitrogen-containing compounds make it difficult for hydroprocessing to be efficiently carried out. In summary, the compounds act as temporary poisons and inhibit hydroprocessing catalysts, they are precursors to coke formation and their hydroprocessing is not economical. Overall, they are one of the biggest hurdles to achieving ultra-low sulfur levels using standard hydroprocessing conditions and catalysts. Intensifying hydroprocessing conditions to overcome some of the challenges is expensive, increases coke formation and affects the quality of the product.

García-Gutiérrez *et al.* (García-Gutiérrez et al., 2014) demonstrated how hydroprocessing significantly improves with the ability to easily obtain ultra-low sulfur diesel after nitrogen-containing compounds were removed from straight run gas oil. They investigated the kinetics of hydroprocessing feedstock of varying nitrogen and sulfur concentrations in straight run gas oil: (0.0020–0.0357 wt%) and (1.3985–1.2130 wt%), respectively, using a commercial CoMoP/γ-Al_2_O_3_ catalyst at 5.49 MPa, liquid hourly space velocity of 2.5 h^−1^, and a temperature range of 622–643 K. The rate of desulfurization varied proportionally with sulfur concentration and inversely with the nitrogen concentration. From their experimental work, they developed a model that showed the sulfur levels that can be achieved as nitrogen levels are varied. The model shows that it is possible to reach 0.0010 wt% sulfur levels if nitrogen compounds are removed prior to hydrodesulfurization. Sau *et al.* (Sau et al., 2005) also showed that hydroprocessing catalyst activity increases by at least 60% and hydrogen consumption is significantly reduced when they carried out a similar investigation of removing nitrogen-containing compounds from straight run gas oil and vacuum gas oil prior to hydroprocessing.

Zeuthen *et al.* (Zeuthen et al., 2001) demonstrated that even though carbazoles are not very reactive and are among the predominant nitrogen compounds, it is the basic nitrogen-containing compounds that play a major inhibitory effect during hydrotreatment of fuel oil. The inhibitory effect of basic nitrogen compounds is magnitudes higher than that of neutral nitrogen compounds while carbazoles are not very inhibiting. Similar studies using different sets of neutral and basic nitrogen compounds are reported throughout literature (Zeuthen et al., 1991; van Looij et al., 1998; Whitehurst et al., 2000; Koltai et al., 2002; Turaga et al., 2003; Wiwel et al., 2010; Hidalgo-Vivas et al., 2022). The behaviour of even trace amounts of basic nitrogen compounds strongly resembles the effect of high concentrations of nitrogen compounds (van Looij et al., 1998; Whitehurst et al., 2000; Zeuthen et al., 2001). The more basic the nitrogen compounds the more the inhibitory effect (Wiwel et al., 2010; Hidalgo-Vivas et al., 2022). Zeuthen and Blom (Zeuthen et al., 1991) characterized the nitrogen on aged hydroprocessing catalysts using temperature-programmed oxidation. They concluded that the nitrogen compounds were attached to the catalyst surface and were not necessarily in the bulk of the coke that was on the surface of the catalyst providing further evidence of the inhibitory effect of the nitrogen compounds on the catalysts.

With that in mind, removing nitrogen-containing compounds (especially the basic ones) as a primary step should improve the efficacy of hydroprocessing and all the other upstream and downstream processes that are affected by these compounds. It is possible that pre-treating to remove nitrogen compounds may be more economical compared to applying more intense hydroprocessing conditions (higher hydrogen pressure, temperature and residence time), improved infrastructure and process design, reduced catalyst lifespan requiring constant replenishment, and obtaining quality product which is not compromized by harsh processing conditions. More importantly, high nitrogen and sulfur content feedstocks such as creosote that are usually shunned by industry can also be pre-treated to remove nitrogen-containing compounds and be economically hydroprocessed to achieve ultra-deep desulfurization. Key to the success of this approach is achieving high selectivity for the nitrogen-containing compounds since the fuel matrix is complex. The fuel matrix is composed of other components (*e.g*. moisture, aromatics, metals and oxygenates) that affect the performance of methods that target nitrogen-containing compounds (Khan et al., 2013; Ahmed and Jhung, 2016a). Some of the compounds in fuel that are important for the quality of the fuel (*e.g.* octane and cetane numbers) also have physichochemical properties close to those of the target nitrogen-containing compounds, *e.g.* aromatics and other heteroatomic compounds (Ghosh et al., 2006). Most approaches can be considered as either *1*) modification of the nitrogen-containing compounds so that extractive or adsorptive techniques can be easily applied or *2*) improvements of the solvents or materials used for extractive or adsorptive separation. Oxidative, extractive, and adsorptive techniques have also been widely researched for desulfurization and the process may also contribute to simultaneous removal of the harsher refractory sulfur compounds. Extraction or adsorption using affordable solvents or materials are more practical approaches and the purified compounds obtained as by-products can also become feedstock for other industries, *e.g*. quinoline and carbazole which are obtained exclusively from coal tar (Fetzner, 1998). We consider it much more beneficial to fine-tune extractive and adsorptive techniques for the selective removal of nitrogen-containing compounds as a complementary technique to hydrodesulfurization.

### Denitrogenation by chemical modification followed by separation

Nitrogen-containing compounds can be derivatized *via* the nitrogen using addition or substitution reagents to precipitate them out of fuel or to make them more polar so that they can be preferentially separated from the rest of the fuel oil using extractive or adsorptive techniques due to their increased relative polarity. Alkylation and oxidation have been extensively investigated in that regard. Alkylating agents usually lead to precipitation. Shiraishi *et al.* (Shiraishi et al., 2001a; Shiraishi et al., 2001b; Shiraishi et al., 2001c) investigated the denitrogenation of aniline, indole and carbazole from xylene (representing light oil feedstocks) using CH_3_I and AgBF_4_ for alkylation. *N-*methylation and subsequent precipitation of the compounds occurred under moderate conditions. The nitrogen content was reduced to less than 20% of the feed concentrations and application on light oil feedstocks was also demonstrated. Bromoacetic acid has been extensively tested for alkylation of nitrogen compounds (Prado et al., 2017). Metallic salts (*e.g.* CuCl_2_, SnCl_2_ and FeCl_3_) have also been used to precipitate basic nitrogen compounds through Lewis acid-base complexation (Prado et al., 2017). *N*-alkylation and precipitation is lucrative as it also simultaneously removes sulfur-containing compounds. However, the method struggles with refractory compounds such as carbazole as electron density on the nitrogen decreases with increasing carbon number of the alkyl substituents. Alkylation of other aromatics is also possible and it is especially difficult to selectively apply the method on high oxygenate content oils.

Catalysts based on strong Lewis acid transition metals (titanium, tungsten, platinum group metals, polyoxometalates, molybdenum and vanadium) have proven to be efficient for the oxidation of nitrogen-containing compounds (Dembaremba et al., 2019a). Various support materials have also been used for the catalysts, *e.g*. silica, alumina, zeolites and organic polymers, and these also have an influence in the performance of the catalysts (Dembaremba et al., 2019a; Dembaremba et al., 2019b). Selective oxidants such as nitric acid and/or nitrogen oxides, organic hydroperoxides, peroxyacids, and hydrogen peroxide are usually employed as oxidants (Dembaremba et al., 2019a; Dembaremba et al., 2019b). Some of the by-products generated by the organic oxidants influence the quality of the fuels; for example, *tert*-butyl alcohol is obtained from *tert*-butylhydroperoxide and improves the octane number (Karas et al., 2008). Hydrogen peroxide is much cheaper but is difficult to use since it is aqueous. Research has been carried out around the introduction of surfactants which help to disperse oxidant in the form of small spherical droplets in fuel oil, a process described as an emulsion catalysis system (Jiang et al., 2011).

Other methods such as autooxidation (using atmospheric oxygen), photochemical oxidation and ultrasound oxidation have also been attempted to cut costs (Shiraishi et al., 2000; Whitehurst et al., 2000). Photochemical oxidation is based on free radical formation and although promising results have been obtained in model samples it is difficult to predict the various reactions that can occur in a complex medium such as fuel oil (Forte et al., 2014). Photodecomposition of nitrogen-containing compounds has been reported by Shiraishi *et al.* (Shiraishi et al., 2000) in model fuel oils with hydrogen peroxide being required in an oil/water biphase to enhance photoreactivity of the nitrogen-containing compounds.

The advantage of the oxidative process is that, unlike hydrotreatment, it operates at lower reaction temperatures and pressure, and in the absence of hydrogen which is expensive. However, large amounts of expensive oxidants are required to completely oxidize the compounds. Our previous work was on improving the catalysts for oxidation in order to reduce the amount of oxidant required but even with good catalysts the amounts of oxidant required still remain high. (Dembaremba et al., 2019a). Using this already expensive process prior to hydrotreatment will result in an expensive product. The capital investment for the additional units is also significant. Product selectivity is also a major challenge (Ishihara et al., 2005). A study by Ogunlaja *et al.* (Ogunlaja et al., 2017) shows the complexity around the oxidation of nitrogen-containing compounds, attributing the array of products to reactions that occur following the formation of hydroxy radicals. Formation of this vast array of products further complicates removal of products after oxidation.

### Extractive denitrogenation

Choice of solvent for the extraction of nitrogen-containing compounds from fuel oils is critical, it must be thermodynamically compatible with the compounds (Dembaremba et al., 2020). Polar solvents with low boiling points such as ethanol, ethanol and acetonitrile have been tested for denitrogenation of fuel oil but these solvents are too volatile for practical application on an industrial scale (Campos-Martin et al., 2010; Mokhtar et al., 2014). Polyalkyleneglycol, polyalkylene glycol ether, imidazolidinones, pyrrolidones, pyrimidinones, dimethyl sulfoxide and dimethyl formamide which have much higher boiling points have shown higher extraction levels of nitrogen-containing compounds (Mokhtar et al., 2014; Abro et al., 2016). However, the boiling points of these solvents are in the same range as some nitrogen-containing compounds, and thus may not be easily separated from the extracted compounds, discouraging solvent recovery by distillation. Besides boiling points, poor selectivity still stands as the major deterrent in the application of solvent extraction.

Considering the poor selectivity witnessed with the use of organic solvents, researchers redirected their efforts towards the use of ionic liquids (ILs) to try and selectively remove nitrogen-containing compounds from fuel. Ionic liquids are described as liquids composed entirely of ions at or below 100°C - more like an ambient temperature molten salt (Abro et al., 2016; Ibrahim et al., 2017). The cations are normally organic and are a basis upon which much of their physicochemical properties are derived, and the anions can be organic or inorganic. Extraction of nitrogen-containing compounds using ILs is based on their solubility that is determined by the chemical interactions involving the acid proton and steric factors in the IL. The use of ionic liquids gained momentum over the past decade with researchers attempting to use various combinations of cations and anions, dialkylimidazolium salts being the most common due to their desirable physical properties and ease of synthesis (Abro et al., 2016).

The chemical structures of ILs are now being tailor made to target certain physicochemical properties to obtain what are termed task specific or designer solvents which allow for better selectivity. In some cases, ILs have been designed to act as both oxidation catalysts and extractants. The ILs catalyse the oxidation of the sulfur- and nitrogen-containing compounds, improving their solubility in the ILs which also act as the extractant (Bhutto et al., 2016). The performance of the ILs in the presence of oxidant is remarkably higher, although efforts are needed to reduce the amounts of oxidant (Abro et al., 2016). Normally polyoxometalates such as vanadium and tungsten are used as the catalytic cations and H_2_O_2_ as oxidant (Lu et al., 2007). Researchers have also explored the use of supported ionic liquid phase (SILP) systems where the ionic liquid is dispersed on a support such as silica to increase surface area, reduce mass transfer limitations and for ease of use (Kuhlmann et al., 2009; Kohler et al., 2010). Leaching is still a challenge, an area that still requires more attention. The development of these SILPs demonstrate the importance of ease of separation in the fuel refinery process which leads use to adsorptive separation (Forte et al., 2014).

Overall, the biggest limit for solvent extraction is poor selectivity. The use of ILs is also mired by the presence of potent complex-forming agents in the fuel matrix. The presence of water, acids, halide ion and other impurities is known to significantly affect their structure and ultimately their physicochemical properties *e.g.*, ILs based on AlCl_3_ and AlCl_4_ form precipitates when exposed to moisture (Dembaremba et al., 2020). The fuel matrix is a complex mixture that contains such components that may incapacitate the ILs over a number of cycles.

### Adsorptive denitrogenation

The use of adsorbents to selectively adsorb nitrogen-containing compounds from fuel oil is the most lucrative option and a lot of research has been revolving around this technique. This is because of the advantages offered by adsorbents that make upscaling feasible and affordable: ease of separation after use, lower risk of noxious sub-products and easy to set-up and operate with the possibility of having a continuous flow system (Putra et al., 2009). However, the challenge has been on finding suitable adsorbents with high capacities and selectivity, and these are dependent on the mechanisms of adsorption.

Adsorption in primitive materials is mainly based on trapping molecules in their cavities based on their sizes (critical diameters and volumes) while in more advanced materials interactive forces that are important for improved selectivity are involved, *e.g*. acid-base interactions, coordination bond formation, π-complexation, hydrogen-bonding and van der Waals forces (Khan et al., 2013; Hasan and Jhung, 2015; Ahmed and Jhung, 2016a). Van der Waals forces are the most popular with hydrogen bonding being more relevant to adsorption of nitrogen-containing compounds. However, van der Waals forces are very weak in nature. Adsorption *via* π-complexation requires the donation of electrons from the π-orbitals of the adsorbates to vacant s-orbitals of the metal and back donation of d-elections of the metal to the empty π antibonding orbitals of the adsorbates. Transition metals in the d-block such as Cu^+^ and Ag^+^ and Ni^2+^ are good candidates for π-complexation, Cu^+^ being more prominent since it is cheaper and more readily available. Basic nitrogen-containing compounds can also undergo a special type of acid-base interaction that forms coordination bonds. The adsorbent serves as the Lewis acid while the nitrogen-containing compounds are the Lewis bases. Based on the Pearson’s hard and soft acid-base theory, basic nitrogen-containing compounds are in the hard to intermediate category and prefer hard Lewis acid sites (*e.g*. Fe^3+^, Cr^3+^, and Al^3+^) (Pearson, 1963). Suitable materials are those with coordinatively unsaturated sites (Kim et al., 2011; Maes et al., 2011; Van de Voorde et al., 2013a).

The drive is now on refining the materials through designing and testing to achieve high selectivity for nitrogen-containing compounds. Research contributions on this subject have been increasing and materials summarized in Table 3 are meant to provide a guide in that regard.

**TABLE 3:** Typical examples of adsorbents that have been tested for adsorptive denitrogenation.

Adsorbent	Feedstock	Performance	References
General adsorbents			
Fe(III) impregnated bentonite clay	Quinoline and methylene blue	Total adsorbed nitrogen (39 mg g^−1^)	Mambrini et al. (2013)
Activated carbon, MAXSORB-II	Straight run gas oil	0.039 g N g adsorbent	Sano et al. (2004)
CuCl/activated carbon	Quinoline and indole in *n*-octane/*p*-xyene (75:25)	AC: quinoline (64 mg/g), indole (63 mg/g); CuCl/AC: quinoline (126 mg/g), indole (168 mg/g)	Ahmed and Jhung, (2015a)
Mesoporous silicas	Light gas oils	Up to 8.05 mg N per g adsorbent	Kwon et al. (2008)
Zeolites containing cuprous cations	Commercial diesel	Alkyl carbazoles completely removed	Hernández-Maldonado and Yang, (2004)
Yttrium ion-exchanged Y zeolite	Indole and quinoline in *n*-octane	Up to 12.37 mg per g adsorbent	Tian et al. (2020)
X-type zeolites	Quinoline in isooctane	Up to 17 mg N per g adsorbent	Ofoghi et al. (2021)
Hexagonal mesoporous silicas (molecular sieves) Ti−HMS	Pyridine, quinoline and indole in *n*-octane (N concentration 200 μg g^−1^) and diesel	Pyridine > quinoline > indole diesel (90% N removal)	Zhang et al. (2010)
Ion exchange resins	Shale-derived oils	Up to 0.072 g N/g resin	Marcelin et al. (1986)
Aluminosilicate mesostructures (MSU-S) and HPW and NiO-HPW modified MSU-S	Quinoline and carbazole in n-hexadecane/*n*-octane (50:50)	MSU-S (0.4 mmol/g), HPW-MSU-S (0.43 mmol/g) and NiO/HPW-MSU-S (0.44 mmol/g)	Rashidi et al. (2015)
		∼7% increase in nitrogen uptake in modified MSU-S	
Tailored organic polymers			
Styrene-divinylbenzene copolymer	Model fuel and crude oil	pyridine (99.9%), pyrrole (99.7%)	Awokoya et al. (2021)
Vinylpyridine based polymer	Indole in *n*-octane	Indole (31.80 mg g^−1^)	Cao et al. (2014)
Polybenzimidazole fibres	Model fuel and spiked diesel	pyrimidine (11.5 mg g^−1^), carbazole (11.8 mg g^−1^), quinoline (11.0 mg g^−1^)	Abdul-quadir et al. (2019)
Poly 4-vinyl aniline-*co*-divinylbenzene	Model fuel and Sasol diesel 500	pyridine (30.2 mg g^−1^)	Mathidala and Ogunlaja, (2019)
Poly-2-(1H-imidazol-2-yl)-4-phenol microspheres	Model fuel and diesel	pyrimidine (10.56 mg g^−1^), carbazole (11.71 mg g^−1^), quinoline (10.84 mg g^−1^)	Abdul-Quadir et al. (2018a)
Poly 2-(1H-imidazol-2-yl)-4-phenol nanofibers	Model fuel and diesel	quinoline (11.7 mg g^−1^), pyrimidine (11.9 mg g^−1^), carbazole (11.3 mg g^−1^)	Abdul-Quadir et al. (2018b)
Fe_3_O_4_ nanoparticles equipped magnetic molecularly imprinted polymers	Model fuel	indole (37.58 mg g^−1^)	Niu et al. (2014)
Coordination polymers			
MIL-101 (Cr)	Straight run gas oil (SRGO) and light cycle oil (LCO)	SRGO (9.0 mg N per g), LCO (19.6 mg N per g)	Nuzhdin et al. (2010)
		Adsorption due to π-π stacking interactions with terephthalate bridges of MOF	
MIL-100 (Al^3+^, Cr^3+^, Fe^3+^, V^3+^)	Indole and 1,2-dimethylindole in heptane	Indole (V>Cr>Fe>Al)	Van de Voorde et al. (2013a)
		1,2-Dimethylindole (V>Cr>Al>Fe)	
		MIL-100(V)vac has best performance due to CUSs	
		Indole>1,2-Dimethylindole	
MIL-101 (Cr)	Pyridine	Pyridine (950 mg/g) pyridine adsorption *via* CUSs	Kim et al. (2011)
MIL-101 (Cr)	SRGO and LCO	MIL-101 (Cr) showed better adsorption than silica gel, Selexsorb^®^ CD, Selexsorb^®^ CDX and activated carbon (2.3 times higher adsorption capacity, two times rate of adsorption)	Laredo et al. (2016)
		Adsorbent regenerated 280 times using acetone	
MIL-100(Fe, Cr, Al), MIL-101(Cr), [Cu_3_(BTC)_2_], CPO-27(Ni), CPO-27(Co), MIL-47/MIL-43	Indole, 2-methylindole, 1,2-dimethylindole in heptane or heptane/toluene (80:20)	No significant uptake (<1 wt%) in MOFs without open metal sites (MIL-47/MIL53)	Maes et al. (2011)
		Reduced uptake when solvent was changed to heptane/toluene (80:20)	
MIL-96(Al), MIL-53(Al) and MIL-101(Cr)	Pyridine, pyrrole, quinoline and indole in *n*-octane	Highest adsorption in MIL-101(Cr) due to CUSs	Wang et al. (2013)
		Adsorption in MIL-96(Al) and MIL-53(Al) demonstrated importance of pore shape and size	
MIL-53(Fe)	Indole and benzothiazole in heptane/isopropanol	Indole (22 wt%), benzothiazole (59 wt%)	Van de Voorde et al. (2013b)
		Hydrogen bonding	
UiO-66—SO_3_H	Indole in *n*-octane	Indole (37% improved uptake compared to pristine UiO-66)	Ahmed et al. (2015)
		Hydrogen bonding with O in (-SO_3_H)	
UiO-66 and UiO-66-NH_2_	Pyridine	Improved adsorption capacity and kinetics in UiO-66-NH2 compared to pristine UiO-66	Hasan et al. (2014)
UiO-66 and UiO-66-NH_2_	Indole	UiO-66 (213 mg/g) > UiO-66-NH2(100) (312 mg/g) due to increased hydrogen bonding from amine group	Ahmed and Jhung, (2015b)
CuCl impregnated MIL-100(Cr)	Quinoline, indole in n-octane/p-xylene (75:25 v/v)	Quinoline (9%) and indole (15%) improved uptake comparedto pristine MIL-100(Cr)	Ahmed and Jhung, (2014)
Phosphotungstic acid impregnated MIL-101	Quinoline, indole	20% increase in quinoline uptake, no change for indole	Ahmed et al. (2013a)
		Acidic MOFs good for the adsorption of hard bases	
AlCl_3_ loaded MIL-100(Fe)	Quinoline and indole	17% increase in uptake of quinoline, no change for indole	Ahmed et al. (2014)
		AlCl_3_ is a Lewis acid salt	
MIL-101(Cr) functionalized with -SO_3_Ag	Quinoline, indole in *n*-octane/toluene (85:15 v/v)	50% increase in uptake, maintained uptake in presence of toluene	She et al. (2018)
Composite materials			
Fe_3_O4@SiO_2_@PILs (magnetic polymeric ionic liquids)	Pyridine, quinoline, indole, carbazole in toluene/heptane (80:20)	Pyridine (80.28%), quinoline (84.45%), indole (32.48), carbazole (28.47)	Wang et al. (2019)
PILs were grafted on silica-coated Fe_3_O_4_			
Mesoporous Ti-HMS/KIL-2 composite	Pyridine and quinoline in *n*-octane	Pyridine(90%), quinoline (90%)	Song et al. (2017)
		Increased surface area compared to precursor compounds	
ZIF-67(x)@H_2_N-MIL-125 [Z67(x)@M125]	Indole, 1-metylindole, quinoline, pyrrole and pyridine in *n*-octane	Indole (680 mg/g)	Bhadra and Jhung, (2020)
		Indole>1-methylindole>quinoline>pyrrole>pyridine	
		H-bonding, cation-π, acid-base and π-complexation	
Graphene oxide (GnO)/MIL-101 (Cr) composite	Indole or quinoline in n-octane	GnO/MIL-101 indole (593 mg/g), quinoline (484 mg/g) > MIL-101 indole (416 mg/g), quinoline (446 mg/g)	Ahmed and Jhung, (2016b)
Graphite oxide/MIL-101(Cr)	Quinoline and indole in *n*-octane/*p*-xylene (75:25)	Improved uptake of quinoline (24%) and indole (30%) in GO/MIL-101 compared to pristine MIL-101	Ahmed et al. (2013b)

#### Adsorptive denitrogenation using traditional adsorbents

Several primitive general adsorbents have been tested as potential adsorbents for removing nitrogen-containing compounds from fuel and these include clays (Mambrini et al., 2013; Baia et al., 2017), silicas and aluminosilicates (Kim et al., 2006; Kwon et al., 2008; Zhang et al., 2010; Rashidi et al., 2015; Laredo et al., 2017; Suganuma et al., 2020), zeolites (Hernández-Maldonado and Yang, 2004; Tian et al., 2020; Ofoghi et al., 2021), activated carbon (Sano et al., 2004; Kim et al., 2006; Ahmed and Jhung, 2015a; Anisuzzamana et al., 2018; Suganuma et al., 2020) and ion exchange resins (Marcelin et al., 1986; XiE et al., 2010) (Table 3). Composite materials that include these materials have also been widely tested (Song et al., 2017; Wang et al., 2019). The biggest advantage is that most of these materials are cheap and readily available. Althought most model fuel studies have yielded wonderful results in terms of loading capacities and recoverability of the adsorbents selectivity remains a challenge when they are applied on real fuel samples. Nevertheless, testing of these materials has provided a lot of information that has inspired the development of various tailor made adsorbents for the removal of nitrogen compounds.

#### Adsorptive denitrogenation using tailored organic polymers

Organic polymers can be effective adsorbents through hydrogen bonding, π-π interactions, van der Waals forces and electrostatic interactions. They can be tactically synthesized to achieve high selectivity for nitrogen-containing compounds through these adsorption mechanisms by creating selective binding sites that recognize and have a higher affinity for template molecules of interest. One approach is to polymerize monomers in the presence of template molecules of interest which are then removed after polymerization through leaching to obtain molecularly imprinted polymers (MIPs) (Awokoya et al., 2021). Several molecularly imprinted polymer materials in different forms (beads, nanofibers *etc.*) have been synthesized and tested for the removal of nitrogen-containing compounds. Typical MIPs and their performances have been summarized in Table 3. (Cao et al., 2014; Niu et al., 2014; Abdul-Quadir et al., 2018a; Abdul-Quadir et al., 2018b; Abdul-quadir et al., 2019; Mathidala and Ogunlaja, 2019; Awokoya et al., 2021). These include Fe_3_O_4_ nanoparticles equipped MIPs that are magnetic for ease of separation (Niu et al., 2014). A method for removing nitrogen compounds from gasoline or diesel fuel using molecularly imprinted polymers has been patented (Rinne and Risto, 2022). Literature search revealed limited study on the use of molecularly imprinted polymer for the selective adsorption of organonitrogen compounds in real fuel samples.

#### Adsorptive denitrogenation using coordination polymers

Research around the refinement of materials to achieve high selectivity has been tending towards coordination polymers (CPs), particularly metal organic frameworks (MOFs) due to the several provisions they offer. CPs are classes of crystalline repeating coordination entities between transition-metal clusters or ions and multidentate organic linkers extending in 1, 2 or 3 dimensions (Kitagawa et al., 2004; Batten et al., 2013). Three-dimensional CPs (MOFs) are the most popular for adsorption work as they contain uniformly sized pores, high total pore volume and very high surface areas (Kitagawa et al., 2004; Jiang, 2015). Due to the ability to tap into both the inorganic and organic aspects, MOFs are ideal candidates for tailoring good selectivity. The design strategies used to obtain particular pore sizes and modify pore properties are also simple, and these include control of their composition, structure, scale and the bulk properties (Schneemann et al., 2014; Kenta, 2017). There is a also a vast range of transition metal clusters/ions and organic building blocks to choose from.

Due to the preferential adsorption of nitrogen-containing compounds, especially on coordinatively unsaturated sites, adsorptive denitrogenation using MOFs is prominent (Ahmed and Jhung, 2016a). Nuzhdin *et al.* (Nuzhdin et al., 2010) pioneered the use of MOFs in adsorptive denitrogenation by testing the performance of the common MIL-101 (Cr^3+^) MOF using indole, carbazole and their alkyl substituted derivatives. They realized an uptake of 9.0 mg nitrogen per gram of MIL-101 from straight run gas oil and 19.6 mg nitrogen per gram of MIL-101 from light cycle oil. They attributed the high sorption capacity to coordination of the nitrogen-containing compounds (*via* nitrogen) to the unsaturated Cr^3+^ centres. Selectivity for nitrogen-containing compounds decreased in the order indole > indole derivatives > carbazole > carbazole derivatives which they explained in terms of indole and derivatives having better steric accessibility of the nitrogen atom to the Cr^3+^ centre while the alkyl substituents cause steric hindrance. Simultaneous adsorption of other aromatic molecules was observed due to π-π stacking interactions with terephthalate bridges of metal-organic framework.

Maes *et al.* (Maes et al., 2011) screened a wide variety of MOFs with and without open metal sites for the selective adsorption of indole (IND), 2-methylindole (2MI), 1,2-dimethylindole (1,2DMI), carbazole (CBZ), and *N*-methyl carbazole (NMC) over TP, BT, and DBT (Table 3). They concluded that the availability and nature of open sites were crucial in the adsorption of nitrogen-containing compounds in the presence of other aromatic compounds. However, uptake was greatly reduced when the solvent system was switched from heptane/toluene (H/T, ratio 80:20 v/v) to toluene/heptane (T/H, 80:20 v/v). The reduction was attributed to the contribution of the aromatic rings in the MOFs and co-adsorption of competing toluene molecules.

Van de Voorde *et al.* (Van de Voorde et al., 2013a) found an Fe terephthlate MOF MIL100(Fe) to be efficient for the selective removal of *N*-heterocyclic compounds (Table 3). They extended their studies to isostructural terephthalate MOFs by varying the metal centre, MIL-100 (Al^3+^, Cr^3+^, Fe^3+^, V^3+^) and investigated the role of the metal centre in the adsorptive removal of nitrogen-containing compounds from fuel. Their calorimetric observations were coherent with the trend in the initial slopes of the isotherms, decreasing in the order: V > Cr > Fe > Al. They confirmed the formation of coordinated species for indole and dimethyl indole. The results for the polarizing power of the Lewis acid centres were inconclusive, similar with most other studies in literature, but it was clear that Fe^3+^ possesses the weakest Lewis acidity in perfect agreement with the observed lack of stable indole-Fe^3+^ coordination complexes in MIL-100(Fe). Al^3+^ in MIL-100(Al) had the lowest affinity for indole. Investigations on the adsorption of pyridine on MIL-101(Cr^3+^) by Kim *et al.* (Kim et al., 2011) also confirmed the formation of a coordination bond between the pyridine N and the coordinatively unsaturated sites in Cr^3+^. Wang *et al.* (Wang et al., 2013) showed that the stronger the basicity of the nitrogen-containing compounds the better the adsorption on MIL-101 (Cr^3+^). They also clarified the importance of pore shape and size in adsorption using MIL-53(Al) and MIL-96(Al). MIL-101(Cr^3+^) which is the best performing MOF in the MIL series was also shown to outperform silica gel, Selexsorb^®^ CD, Selexsorb^®^ CDX and activated carbon by far in the adsorption of nitrogen-containing compounds from straight run gas and light cycle oil with exceptional adsorption capacity and the fastest kinetics (Laredo et al., 2016). Adsorption studies on IL-53(Fe) which contains no coordinatively unsaturated metal sites showed that the material relies on adsorbates being good hydrogen bond acceptors (Van de Voorde et al., 2013b).

**FIGURE 5:**
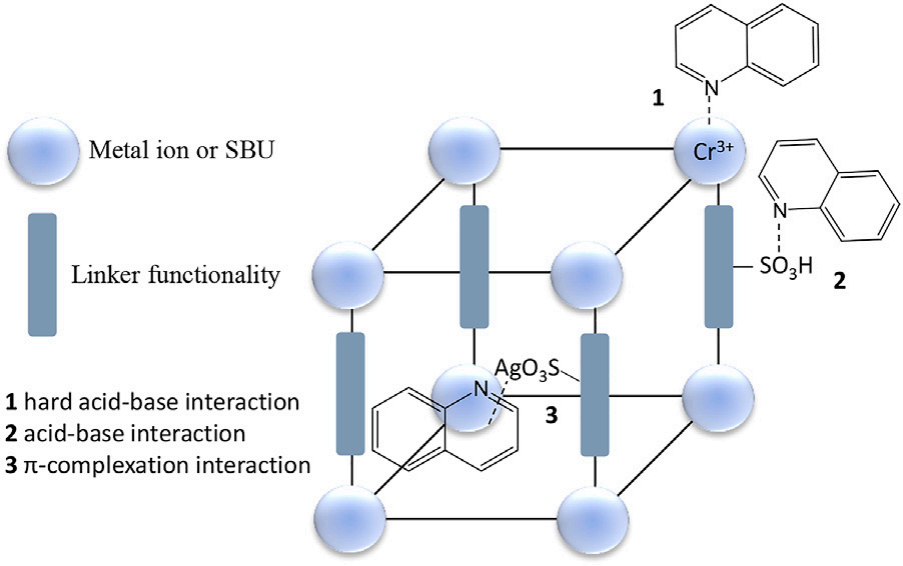
Schematic representation of the multiple interaction sites in the highly selective MOF, (Cr)-MIL-101-SO_3_Ag, reported by She *et al.* (She et al., 2018).

Following these revelations on what matters regarding adsorption of nitrogen-containing compounds using MOFs researchers went on a drive to improve the adsorption performance of other MOFs through modifications which include incorporation of functional groups (*e.g*. –SO_3_H, amino group) (Hasan et al., 2014; Ahmed et al., 2015; Ahmed and Jhung, 2015b) and Lewis acid sites (*e.g*. CuCl, phosphotungstic acid and AlCl_3_) (Ahmed et al., 2013a; Ahmed et al., 2014; Ahmed and Jhung, 2014) (Table 3). Composites between MOFs, and MOFs with other materials such as graphene oxide have also been tested (Table 3) (Ahmed et al., 2013b; Ahmed and Jhung, 2016b; Bhadra and Jhung, 2020). Lewis acid sites are also used to add functional moieties inside the pores of the MOFs (Van de Voorde et al., 2013b). She *et al.* (She et al., 2018) reported a highly selective metal organic framework with immobilized Ag(I) sites, (Cr)-MIL-101-SO_3_Ag, which nitrogen-containing compounds to interact with adsorption sites through multiple ways simultaneously (Figure 5).

The approach of using MOFs is not impeccable. Firstly, MOFs are generally expensive due to the types of ligands or synthesis protocols used to obtain them. It can be capital intensive in industries such as the fuel industry where bulk quantities are required. In that regard, development of MOFs is being carried out with ease of separation and reusability in mind. Magnetism has been applied in some MOFs for ease of separation from the fuel matrix (Jin et al., 2014). Carrying out adsorption under continuous flow conditions can also be helpful. Secondly, synthesizing the desired MOF in a short period of time when scaling up remains a challenge. Sometimes the bulk powder form does not always match the structure of the target product. Bulk quantities are also usually obtained in a fine powder form with poor mechanical strength which makes re-use difficult. Aggregation during use or recycling may compromise the performance of the MOFs. Thirdly, some MOFs are sensitive to the environment, *e.g*., temperature and moisture. Lastly, adsorption in MOFs is based on small pore apertures which result in high diffusion resistance due to steric and dynamic hindrance limiting the movement of sulfur- and nitrogen-containing compounds into the inner pores. Nevertheless, if adsorptive denitrogenation is to be achieved it will most likely be through coordination polymer based-materials due to the several advantages they offer.

## Perspectives and advances

Hydrotreament remains the main process used for removing sulfur- and nitrogen-containing compounds from crude oil. It is easier for industry to adopt improvements around this process compared to introducing new processes. The limits of the technique (remaining economically viable and the ability to process harsh feedstocks) are now being challenged by the recently imposed legislations that require ultra-low sulfur levels (<10 ppm) in fuel oil products. This has prompted major reconsiderations into process optimizations, catalyst designs, use of additives, blending with cleaner streams, and possible complimentary or alternative desulfurization methods. To date, none of the proposed alternative desulfurization methods such as biodesulfurization, extractive desulfurization, oxidative desulfurization and adsorptive desulfurization has resulted in a commercially viable process. The main inhibiting factor is the additional processing units required to incorporate the alternative technologies, such as the oxidation unit and extraction unit which require a high capital investment. It may be cheaper to add an adsorptive denitrogenation pre-treatment step since pre-treatment steps already exist in industry for example towards demulsifying, desalting, deasphalting and demetallization.

Improvements around the hydroprocessing technique has reached its apex, especially process optimization, and major changes will be difficult to achieve unless fundamental changes are made to the catalysts. There are still gaps that can be addressed in the design and use of catalysts, an area that was extensively covered in this review. Catalyst improvements range from perfecting the support materials, fine-tuning the active phases of standard catalysts by using chelating agents and additives, design of PMs-containing catalysts and trimetallic systems, and other synthesis strategies. From the works cited, catalyst activity improves with these changes. However, the efficiency of the improved catalysts on refractory compounds still remains a challenge. Even with those improvements, the catalysts are still not immune to the inhibitory effects that mostly come of nitrogen containing compounds. The inhibitory effects of nitrogen-containing compounds also have a serious impact on achieving ultra-deep hydrodesulfurization. As a result, there is huge motivation for pre-treatment of feedstocks to remove nitrogen-containing compounds prior to hydroprocessing in order to achieve deep hydrotreatment.

Despite the lack of industrial application of adsorbents currently for removal of nitrogen-containing compounds from crude oil, research in this direction is promising. Several strides have been made in the refinement of liquid-liquid partitioning techniques, with ionic liquids addressing most of the challenges that limit the selectivity of extraction solvents. The use of traditional adsorbents such as zeolites and carbon materials suffer lack of specificity for the nitrogen-containing compounds but nanoarchitectured coordination polymers (CPs) have recently been overcoming most of the challenges. It was identified that lack of synergy among the key features during adsorption, especially the metal centre and linker properties, is the biggest shortfall in most of the CPs from achieving good selectivity. It is assumed that an entity is either adsorbed based on its interaction with the linker or through interaction with the metal centre, but not effectively with both at the same time. That could also explain the lack of coherence in the results reported by authors so far with change in linkers, target molecules and when toluene was added as no resolute trend can be interpreted from the data. It was also noted that when looked at independently, coordination *via* coordinatively unsaturated sites provides a better chance to achieve good selectivity compared to any other mechanism. As considerations for the key features to work together are being made, it is important to have coordinatively unsaturated sites as the primary focus and exclude the other adsorption mechanisms as much as possible if good selectivity is to be achieved. This is particularly more important for crude oil since it is a very complex mixture with several competing molecules. Recent work from She *et al.* (Ahmed et al., 2015) has clearly demonstrated how focusing on design strategies can lead to highly remarkable selectivity for nitrogen-containing compounds. The governing factors should also be affordability and reusability of the coordination polymers.

Pre-treatment of crude oil to remove nitrogen-containing compounds undoubtedly helps to achieve ultra-deep hydrodesulfurization using conventional hydroprocessing. Coupled with the successes being made with coordination polymers, as well as as other adsorbents and solvents for liquid-liquid partitioning, it warrants research to put more effort in this direction. Removal of nitrogen-containing compounds from feedstock can also result in significant improvements of other downstream processes. High nitrogen content feedstock such as creosote are becoming more readily available syncrude oil sources and this approach will make it possible to process them sustainably. Besides, hydrodenitrogenation in itself is not an economical process hence pre-treatment of feedstock to remove nitrogen-containing compounds should improve the efficiency of hydroprocessing and also reduce operational costs. With concerted efforts on adsorbent and catayst development/catalyst stacking technology, it should be possible to study the application of these techniques in tandem for ultra-deep desulfurization. It will be important to consider the economical aspects for the implementation of this technique, of which there is no literature that clearly provides answers in that regard. It is crucial to provide more literature on the total cost and benefits of adding the pre-treatment step compared to intensifying hydroprocessing conditions and continuously replacing catalyst.
